# Characteristics, Diagnosis and Prognosis of Acute-on-Chronic Liver Failure in Cirrhosis Associated to Hepatitis B.

**DOI:** 10.1038/srep25487

**Published:** 2016-05-05

**Authors:** Hai Li, Liu-Ying Chen, Nan-nan Zhang, Shu-Ting Li, Bo Zeng, Marco Pavesi, Àlex Amorós, Rajeshwar P Mookerjee, Qian Xia, Feng Xue, Xiong Ma, Jing Hua, Li Sheng, De-kai Qiu, Qing Xie, Graham R Foster, Geoffrey Dusheiko, Richard Moreau, Pere Gines, Vicente Arroyo, Rajiv Jalan

**Affiliations:** 1 Department of Gastroenterology, Ren Ji Hospital, School of Medicine, Shanghai Jiao Tong University, Shanghai, China; 2 Shanghai Institute of Digestive Disease, Shanghai, China; 3 Key Laboratory of Gastroenterology & Hepatology, Chinese Ministry of Health (Shanghai Jiao Tong University), Shanghai, China; 4 Data Management Centre, European Foundation for the study of Chronic LIver Failure (EF-CLIF), Barcelona, Spain; 5 Department of Liver Surgery and Liver Transplantation, Ren Ji Hospital, School of Medicine, Shanghai Jiao Tong University, Shanghai, China; 6 Department of Infectious Disease, Rui Jin Hospital, School of Medicine, Shanghai Jiao Tong University, Shanghai, China; 7 Queen Mary’s University of London, Barts Health, United Kingdom; 8 Service d’Hepatologie, Hopital Beaujon, Clichy, France; 9 Liver Unit, Hospital Clinic, Barcelona, Spain; 10 European Foundation for the study of Chronic Liver Failure (EF-CLIF), Barcelona, Spain; 11 Liver Failure Group, Institute for Liver and Digestive Health, UCL, London, United Kingdom

## Abstract

The diagnostic and prognostic criteria of acute-on-chronic liver failure (ACLF) were developed in patients with no Hepatitis B virus (HBV) cirrhosis (CANONIC study). The aims of this study were to evaluate whether the diagnostic (CLIF-C organ failure score; CLIF-C OFs) criteria can be used to classify patients; and the prognostic score (CLIF-C ACLF score) could be used to provide prognostic information in HBV cirrhotic patients with ACLF. 890 HBV associated cirrhotic patients with acute decompensation (AD) were enrolled. Using the CLIF-C OFs, 33.7% (300 patients) were diagnosed as ACLF. ACLF was more common in the younger patients and in those with no previous history of decompensation. The most common organ failures were ‘hepatic’ and ‘coagulation’. As in the CANONIC study, 90-day mortality was extremely low in the non-ACLF patients compared with ACLF patients (4.6% vs 50%, p < 0.0001). ACLF grade and white cell count, were independent predictors of mortality. CLIF-C ACLFs accurately predicted short-term mortality, significantly better than the MELDs and a disease specific score generated for the HBV patients. Current study indicates that ACLF is a clinically and pathophysiology distinct even in HBV patients. Consequently, diagnostic criteria, prognostic scores and probably the management of ACLF should base on similar principles.

Acute on chronic liver failure (ACLF) is a dynamic syndrome observed in the hospitalized cirrhotic patients either with or without an identified precipitating factor characterized by hepatic and or extrahepatic organ failures and high mortality^1^. The elements of the above working definition was proposed by a working group under the auspices of the European and the America association for the study of liver (European Association for the Study of the Liver/the America association for the study of liver Disease, EASL/AASLD) in 2010^2^. Since then, large prospective studies were performed by the EASL chronic liver failure (CLIF) Consortium, the data from which suggested that the diagnosis of ACLF can be accurately made using the CLIF-Consortium Organ Failure score (CLIF-C OFs; CLIF Acute-on Chronic Liver Failure in cirrhosis (CANONIC) study) and its prognosis determined using the CLIF-Consortium acute-on chronic liver failure score (CLIF-C ACLFs)^3^,^4^. In patients with acute decompensation^4^ (AD, refers to the acute development within 1-month before admission of ascites, variceal hemorrhage, hepatic encephalopathy and/or bacterial infections) who did not fulfill criteria for the diagnosis of ACLF, the CLIF acute decompensation score (CLIF-Consortium acute decompensation score, CLIF-C ADs) was validated to provide prognostic information^5^. The North American Consortium for the Study of End Stage Liver Disease (NACSELD), made similar observations confirming high mortality rates of patients with organ failures in the hospitalized cirrhotic patients with bacterial infection^6^.

One of the limitations of the CLIF consortium classification is that it has only been validated in cohorts of patients from Europe and the patient populations studied had an extremely low prevalence of HBV infection, which is the commonest cause of chronic liver disease in the Eastern Pacific rim. Given that infection with HBV may be associated with disease flares, which can lead to rapid deterioration in liver function despite introduction of effective antiviral therapy, it is unclear whether criteria developed for non-HBV induced ACLF can also be applied to patients with HBV related ACLF. A Consensus definition has of necessity been developed under the auspices of the Asia Pacific association for the Study of liver disease for the definition of ACLF, which is however, based on expert opinion, rather than prospectively validated data. The definition proposed by the Asia-Pacific association for the study of liver Disease (APASL) was “acute hepatic insult manifesting as jaundice and coagulopathy, complicated within four weeks by ascites and/or encephalopathy in a patient with previously diagnosed or undiagnosed chronic liver disease^7^”. As is self evident, the proposed definitions are very different^8^. Therefore, a World Consensus on the definition of ACLF was organized under the auspices of the World Congress of Gastroenterology, which amongst other recommendations concluded that the CLIF criteria should be validated in an Asian population that included patients with HBV infection^9^.

Therefore, the aims of this study was to evaluate whether the CLIF-C OF score can be used to classify patients presenting with HBV infection and AD into an ACLF and no ACLF groups with distinct clinical characteristics and outcomes. We then determined whether the CLIF-C ACLF and the CLIF-C AD scores provide prognostic information in these two patient groups in comparison with standard prognostic scores. The study also aimed to develop a HBV specific score and compare its performance with the CLIF scores. In order to achieve these aims, the EASL CLIF Consortium set up collaboration with Ren Ji Hospital, Shanghai, China and all the data were analyzed by the Data Management Centre of the EASL CLIF Consortium in a manner similar to that performed for the CANONIC study^4^ to allow direct comparison.

## Results

### Clinical characteristics of the patients at the time of hospital admission

**Figure 1:**
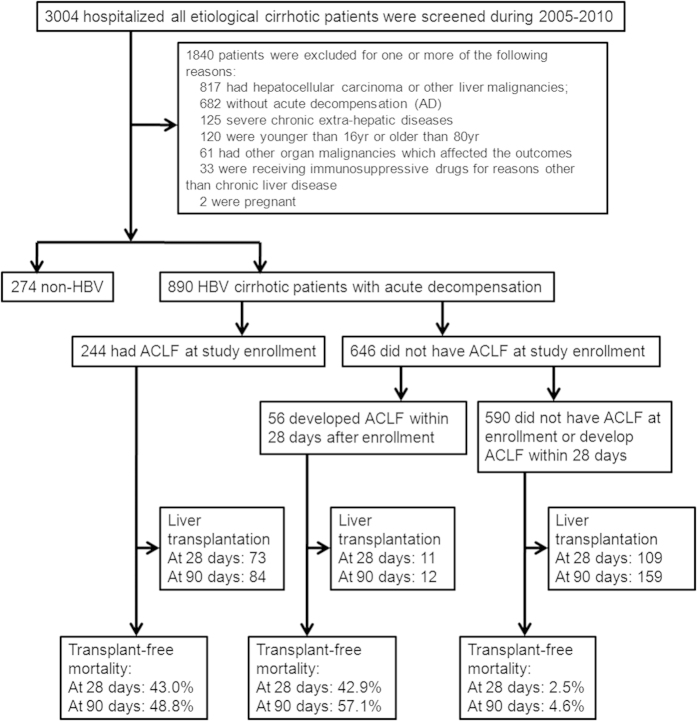
Screening, enrollment, and flow of patients according to the presence or absence of ACLF according to CLIF-C OF score.

The flow chart for both screening and enrollment of patients was presented at Fig. 1. There were 3004 hospitalized cirrhotic patients screened in a single tertiary hospital (Ren Ji Hospital) in Shanghai from January 2005 to December 2010 and 890 HBV associated cirrhotic patients with AD were enrolled. Clinical characteristics of enrolled patients are summarized in Supplementary table S1. The mean age in the current series was 50 years and most patients (76.7%) were male. Seven-hundred and thirty-three patients (82.4%) presented significant HBV replication (HBV-DNA > 100 IU/ml) in concordance to the low percentage of patients (23.5%) treated with nucleotide analogs (NUCs) within 6 months prior hospitalization. Most patients had received lamivudine and none had been treated with tenofovir (a NUC with a high barrier to resistance). In 274 patients, NUCs were started after hospital admission with AD. Prior history of AD was present in only 55.1% of patients. In the vast majority of patients (90.7%) no other cause for cirrhosis other than chronic HBV was found. In the remainder patients HBV infection was associated with alcoholism (6.2%), schistosomiasis (1.2%) and other hepatic viruses (1.9%; HCV 0.8%, HEV 0.8% and CMV 0.7%).

The main indication for hospital admission was ascites (56.9%) followed by GI-bleeding (26.5%), hepatic encephalopathy (11.8%) and bacterial infections (4.4%). Potential precipitating events of acute decompensation were present in 54.8% (488/890) patients. The most frequent precipitating event (PE) was bacterial infection (10.6%), followed by GI-bleeding and active alcoholism (7.5% and 7.2%, respectively). HBV reactivation was observed in only 41 patients (4.6%). In 7 patients, this was related to sudden cessation of anti-viral therapy. No cases of *de novo* reactivation were observed. Only 99 patients (11.1%) presented more than one PE.

Three hundred and eighty patients (42.7%) presented at least one OF. The most frequent OF was hepatic failure, which was present in 30.1% of the patients followed by coagulation failure (26.1%), renal failure (10.0%), cerebral failure (5.2%), circulatory failure (2.4%) and respiratory failure (1.2%). Renal and cerebral dysfunctions were present in 5.3% and 8.3% of patients, respectively. Mean model for end-stage liver disease (MELD) and MELD-Sodium (MELD-Na) scores were 20.2 and 22.6, respectively.

Two hundred and forty-three patients (27.3%) presented with ACLF at admission: 45 patients (5.1%) had ACLF-1, 132 (14.8%) ACLF-2 and 66 (7.4%) ACLF-3.

### Clinical characteristics of patients with ACLF at admission or developing it during hospitalization

In addition to the 243 patients presenting with ACLF at admission, 57 additional patients (6.3%) developed ACLF during hospitalization. Three hundred patients, therefore, had ACLF during the study period, which represents a prevalence of 33.7%. Fifty-five patients (6.2%) had ACLF-1, 147 (16.5%) ACLF-2 and 98 (11.0%) had ACLF-3.

Table 1 shows the characteristics of patients with (300) and without (590) ACLF during the study period. Data in patients with ACLF were obtained at the time of ACLF diagnosis (at admission or during hospitalization). The most common OF in patients with ACLF was liver failure (77.7%), followed by coagulation failure (67.7%), renal failure (28.3%), cerebral failure (23.7%), circulatory failure (19%) and respiratory failure (14.3%). The prevalence rates of renal and cerebral dysfunction in patients with ACLF were 14.6% and 18.0%, respectively. The prevalence of single non-renal organ failure in patients without ACLF was extremely low; liver failure in 7.8%, coagulation failure in 9.8%, cerebral failure in 0.5% and circulatory failure in 0.3%. Renal failure by definition is ACLF and no patient without ACLF showed respiratory failure. Renal and/or cerebral dysfunctions were also very infrequent in patients without ACLF (0.4% and 2.9%, respectively).

Patients with ACLF were younger; more frequently had no prior AD episodes and had been more frequently treated with NUCs within the 6 months prior to admission than patients with AD without ACLF. The choice of therapy did not impact upon the development of ACLF and, importantly, there was no significant difference in the proportion of patients with ACLF receiving the low resistance barrier drug lamivudine compared to the high resistance barrier agent entecavir. Bacterial infections, active alcoholism, HBV reactivation and superimposed viral infections were more frequent in patients with ACLF than in those without ACLF. In contrast, there was no difference in GI-bleeding, hepatotoxic drugs/herbs and surgery between groups. Accordingly, the prevalence of 1 or more PE was more frequent in patients with ACLF. Patients with ACLF showed higher serum concentration of serum aminotransferase and lower mean arterial pressure and serum concentration of sodium than patients without ACLF.

**Figure 2:**
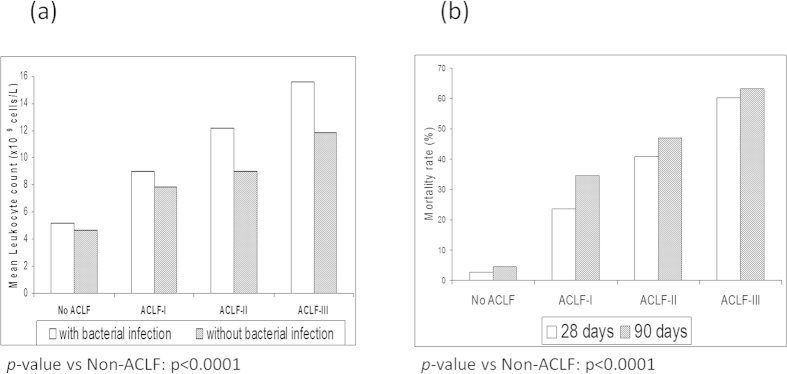
(**A**) Relationship between the severity of ACLF and the WCC.(**B**) Mortality rate at 28 days and 90 days according to the grade of ACLF.

White blood cell counts (WCC) were significantly higher in patients with ACLF than in those without ACLF. This difference was observed in patients with bacterial infections (5.2 ± 2.6 vs. 12.9 ± 9.9 × 10^9^ cells/l; p < 0.01) as well as in patients without bacterial infections (4.7 ± 3.3 vs. 9.7 ± 5.7 × 10^9^ cells/l; p < 0.01). Figure 2A shows that there was a close direct relationship between the WCC and the grade of severity of ACLF.

### Factors predicting the development of ACLF during hospitalization

Univariate analysis was performed using data obtained at admission in patients without ACLF who did or did not develop ACLF during hospitalization (Table 2). Presence of PE’s, bacterial infections and liver failure at admission were significantly more frequent, WCC, international normalized ratio (INR), alanineaminotransferase (ALT) and aspartate aminotransferase (AST) significantly higher and hematocrit and serum sodium significantly lower in patients developing ACLF during hospitalization than in those not developing the syndrome. Multivariate analysis disclosed leukocyte count (OR = 1.15; 95%CI: 1.08–1.24; p < 0,001), bilirubin (OR = 1.05; 95%CI: 1.02–1.07; p = 0,002), INR (OR = 1.66; 95%CI: 1.10–2.51; p = 0,016) and bacterial infection (OR = 3.62; 95%CI: 1.53–8.59; p = 0,004) as independent predictors of ACLF during hospitalization. There were no statistically significant differences for HBV reactivation and the level of HBV DNA between ACLF and non-ACLF groups in the univariate and multivariate analysis.

### Mortality

**Figure 3:**
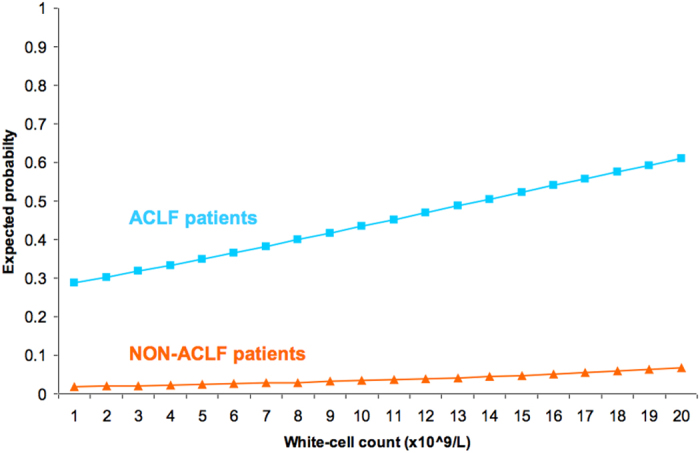
Relationship between the expected probability of death at 28-days, the presence or the absence of ACLF and the white cell count.

The 28-day and 90-day mortality rates were extremely low (2.6% and 4.6%, respectively) in patients without ACLF and very high (44% and 50%, respectively) in patients with ACLF. Following the first six-month period, mortality rates did not increase significantly in both groups (Table 1). Figure 2B shows that there was a close direct relationship between the severity of ACLF grade at diagnosis and the 28-day and 90-day mortality rates in patients with ACLF. The probability of death in patients with and without ACLF increased with the white-cell count (Fig. 3). However, for any given value of white-cell count, the expected death rate was significantly higher in ACLF patients. In total, 177 patients died at 90 days. The main cause of death was ACLF (125, 70.7%), followed by hypovolemic shock (24, 13.5%), septic shock (24, 13.5%) and other/unknown (4, 2.3%).

**Figure 4:**
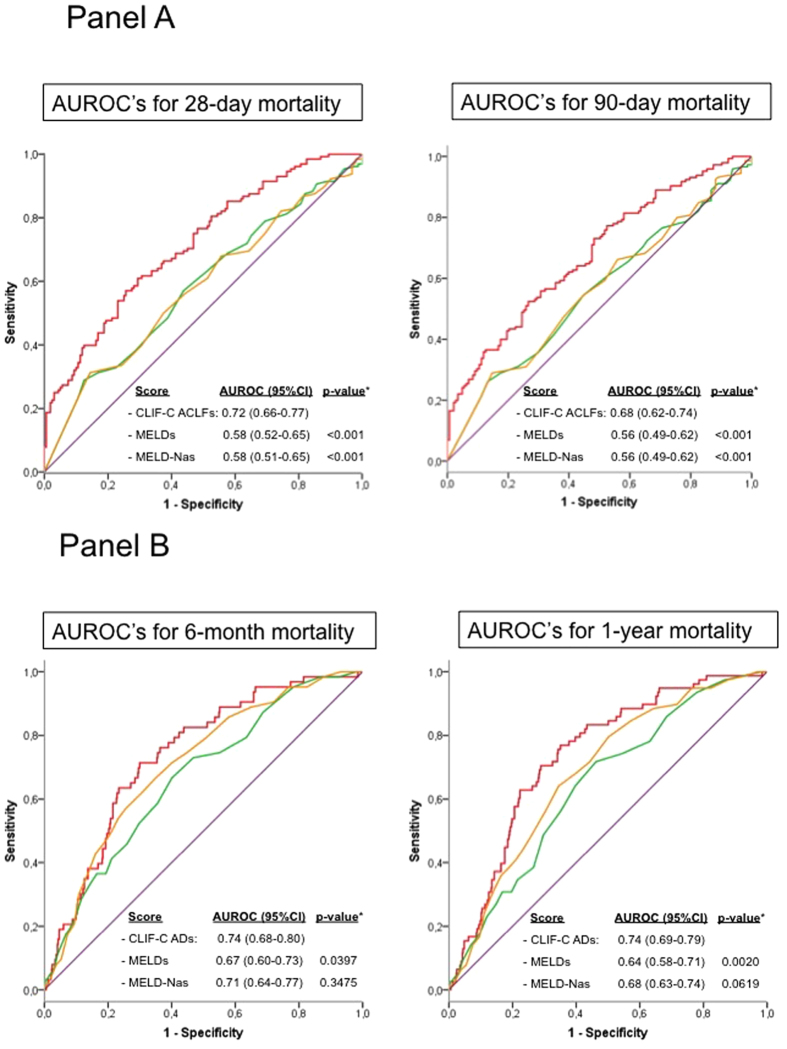
Panel (**A**): Accuracy of the CLIF-C ACLFs (red line) as compared to MELDs (green) and MELD-Nas (orange) in predicting 28-day and 90-day mortality of patients with ACLF associated to cirrhosis due to HBV infection. Comparison of the areas under the ROC curves (AUROCs) estimated for each score. The CLIF-C ACLFs showed a significantly higher predictive ability in comparison with the MELDs and MELD-Nas scores for both the 28-day and 90-day mortality. Panel (**B**): Accuracy of the CLIF-C ADs (red line) as compared to MELDs (green) and MELD-Nas (orange) in predicting 6-month and 1-year mortality of patients with AD (without ACLD) due to HBV associated cirrhosis. Comparison of the AUROCs estimated for each score. The CLIF-C ADs showed significantly higher predictive ability in comparison to the MELDs.

The CLIF-C ACLF score (Fig. 4, Panel A) and the CLIF-C AD score (Fig. 4, Panel B) were significantly more accurate than the MELD and MELD-Na scores in predicting short term and long term mortality in patients with and without ACLF, respectively.

To assess whether specific prognostic scores for patients with HBV associated cirrhosis could increase the accuracy of CLIF-C ADs and CLIF-C ACLFs, two new scoring systems, the HBV-ACLFs and the HBV-ADs, were developed based on the best predictors of survival in our patients with and without ACLF. In univariate analyses age, white-cell count and creatinine were found to be significantly associated with 28-day mortality in ACLF patients, while age, bilirubin, white-cell count, INR, creatinine and serum sodium significantly predicted 1-year mortality in non-ACLF patients. The derived score coefficients were based on the best sub-sets of predictors selected in the corresponding survival models:

*HBV-ADs* = *0.041 age* + *0.270 log (serum bilirubin)* + *0.82 log (WCC)*

*HBV-ACLFs* = *0.020 age* + *0.256 log (serum creatinine)* + *0.709 log (WCC)*

Both scores improved the accuracy of MELD and MELD-sodium but not the accuracy of CLIF-C ADs and CLIF-C ACLFs (Table 3). In fact, CLIF-C ACLFs was significantly more accurate than the HBV-ACLFs at all main end-points.

### Differences between ACLF in HBV associated cirrhosis and ACLF in cirrhosis due to other etiologies included in the CANONIC study^4^

Results obtained in the current study in patients with ACLF associated to HBV infection (HBV-ACLF) were compared to those previously reported by the CANONIC Study in patients with ACLF associated with cirrhosis of other etiologies (CANONIC-ACLF) (Table 4). Age was significantly lower (46.5 (11.3) vs. 55.8 (11.7); p < 0.001) and male gender significantly more frequent in patients with HBV-ACLF, but these features were also observed in patients with AD without ACLF (data not shown). The prevalence of liver failure and coagulation failure was significantly higher and the prevalence of renal failure was significantly lower in HBV-ACLF. There were no differences in the prevalence of other OFs between groups. Accordingly, liver function tests (serum bilirubin, ALT and AST, and INR) were significantly worse in patients with HBV-ACLF infection while serum creatinine, gamma-glutamyltranferase and serum sodium, were significantly higher in patients with CANONIC-ACLF. There were no differences in WCC between groups. Importantly, the incidence of a prior history of AD was significantly lower in patients with HBV-ACLF indicating that a higher proportion of patients developed ACLF at the same time as the first AD of cirrhosis.

The prevalence of ACLF-1 was significantly higher in CANONIC-ACLF. In contrast, the prevalence of ACLF-2 and ACLF-3 was significantly more frequent in patients with HBV-ACLF. Accordingly, the 28-day and 90-day mortality was significantly higher in patients with HBV-ACLF.

## Discussion

The results of this study validate the diagnostic ability of CLIF-C OFs to differentiate hospitalized HBV patients with decompensated cirrhosis into those with ACLF and those without. As observed in the CANONIC study^4^, the 28-day mortality rate for our patients without ACLF was very low (2.6%) compared with a 44% mortality in those classified as ACLF. Moreover, the data demonstrated the significance of OFs in determining survival in this HBV population; mortality rates increased progressively with the number of OFs. The CLIF-C ACLF and the CLIF-C AD scores performed better at predicting prognosis than MELD and MELD-Na scores^3^,^5^. Specific score developed from the data of patients with HBV associated ACLF (HBV-ACLFs) was less accurate than the previously defined CLIF-C ACLF score in predicting outcome. From the pathophysiological perspective, the putative importance of inflammation in the pathogenesis of ACLF was confirmed in the HBV population, as white cell count correlated with the presence and grade of ACLF and remained an independent predictor of mortality^4^. Taken together, the data suggest that the CLIF Consortium criteria for the diagnosis and prognosis of hospitalized cirrhotic patients that was developed in Europe in non-HBV patients can also be used in Chinese patients with HBV infection with a high degree of accuracy.

Despite the many similarities in the diagnostic and prognostic criteria for ACLF in HBV patients with the CANONIC cohort, important differences were observed in the Chinese cohort with HBV compared with the European non-HBV patients suggesting that ACLF due to hepatitis B confers unique characteristics to the syndrome.

One of the most intriguing observations was the main organ failures in HBV cohort was ‘Liver’ and ‘Coagulation’, which tended to occur together and more frequently (78.7 and 68.4% respectively) accounting for ACLF gradation of ACLF-2. As the proportion of patients with ACLF-2 was over represented while the proportion of patients in the ACLF-1 cohort was low in the HBV population, the 28-days mortality of the HBV cohort was significantly higher than that observed in the non-HBV, CANONIC cohort (43% vs. 30%)^4^. A possible explanation for the observation of increased ‘Liver’ and ‘Coagulation’ failure is likely to be the pathological characteristics of HBV ACLF patients. Our previous study^10^ explored that submassive hepatic necrosis accompanied with cholestasis and/or ductular bilirubinostasis (sepsis) were the major hepatic pathological features of HBV related ACLF patients in contrast to mainly inflammation and bilirubinostasis in the alcohol related ACLF patients^11^,^12^. Lacking prothrombin biosynthesis caused by extensive parenchymal hepatocyte destruction and severe cholestasis around residual cirrhotic nodules in HBV submassive hepatic necrosis liver are, therefore, reasonable explanations for prolonged INR and hyperbilirubinemia. For precipitating events, more than 80% patients were not receiving NUC’s and therefore, it was not surprising to note that they had detectable levels of HBV DNA at the time of hospital admission but surprisingly, less than 5% patients showed clinical and virological evidence of HBV reactivation^13^. There was no significant difference in the distribution of HBV-DNA levels in patients who did, or did not, develop ACLF suggesting that HBV associated hepatitis was not related to the development of liver dysfunction^14^,^15^. However it is well established that changes in HBV-DNA may precede liver injury and given that the transaminase levels were increased in patients with ACLF compared to those who did not develop ACLF it is impossible to exclude an effect of HBV. As no other precipitating event was detected in about 60% of the patients, it is possible that there may well have been ‘HBV flares’ accounting for the development of ACLF but this hypothesis will need to be studied further^16^,^17^. It was also notable that other risk factors for ACLF such as infection with other hepatitis viruses were over represented in those HBV patients that developed ACLF. Not surprisingly, alcohol abuse on the background of HBV cirrhosis was also associated with the occurrence of ACLF more frequently. As in the CANONIC cohort, the clinical course of ACLF and the associated mortality were not related to the presence and type of precipitating events. This indicates that, although precipitating events are important in the development of ACLF, once it develops the clinical course and mortality depend more on the severity of systemic inflammation and number of OFs.

Another interesting observation was that, in contrast to the CANONIC study, which comprised non-HBV patients, kidney failure rate in the HBV patients was significantly lower (52% vs. 28.6%). The mechanism of lower rates of renal failure in the HBV ACLF population is not clear but may suggest different mechanisms of injury. In the alcoholic cirrhosis population, which formed the majority of patients in the CANONIC study, gut bacterial translocation is pathophysiologically important and has been shown to possibly ‘prime’ the kidneys by up-regulation of toll-like receptor 4 to the effect of a superimposed inflammatory insult^18^,^19^,^20^. Alternatively, or in addition, this difference may reflect that a ‘hepatic’ insult such as HBV flares/reactivation compared with ‘extrahepatic’ insult such as bacterial infection or alcohol abuse, which were the predominant precipitating illnesses in the CANONIC cohort^4^, was an important mechanism of ACLF in our series. It is therefore, interesting to note that in HBV patients with infections or alcoholism as the precipitating event, the rate of renal failure was 19.6%, (30/153 patients), which was significantly higher than in patients without these PE’s (7.5% (55/737) patients) (P < 0.001).

It is also interesting to note that white cell count was an independent predictor of mortality in both the cohorts indicating the strong and the very important influence of inflammation in driving the pathogenesis of HBV related ACLF, as was the case in the CANONIC patients. As illustrated in Fig. 2A, the white cell count was significantly higher in all the ACLF cohorts and increased progressively with more severe grades of ACLF. Remarkably, the presence of bacterial infection at each stage of ACLF was associated with markedly higher WCC count, which was absent in the non-ACLF cohort. Moreover, as illustrated in Fig.3, the correlation between leucocyte count and 28-day mortality showed that for any given value of white-cell count and (presumably) inflammation, the probability of death was significantly higher in patients with ACLF than in those without the syndrome. Bacterial infection as a precipitating event was significantly more commonly associated with ACLF and its presence was associated with more severe grades of ACLF in this HBV cohort as was the case with the CANONIC patients^4^. These observations, which confirm the results from the CANONIC study, suggest that in addition to systemic inflammation, altered host response to injury accounts for organ failure even in the HBV patients who develop ACLF^1^. Further studies are needed to define the underlying mechanisms.

HBV patients with ACLF were significantly younger than patients in the European cohort but in both cohorts, the ACLF patients were younger than the patients with AD. Severity of ACLF also correlated inversely with age. Additionally, 52.3% patients with HBV related ACLF had no previous history of decompensation, a proportion that was significantly higher than in patients with HBV infections without ACLF. Taken together, the data show clearly that HBV-ACLF is pathophysiologically similar to the CANONIC patients as in both cohorts ACLF was more commonly associated with younger age, lack of previous decompensation and inflammation^21^,^22^.

The CLIF-C ACLF score was developed to provide sequential prognostic information in ACLF patients in the non-HBV cohort and validated independently in a similar population^3^. Its performance in predicting mortality was shown to be of similar accuracy in the HBV-ACLF patients. Importantly, its performance was significantly better than the best scoring systems that are currently used to allocate organs for liver transplantation, the MELD^23^ and the MELD-Na^21^ scores providing a further independent validation of the CLIF-C ACLF score for HBV-ACLF patients. Using the dataset, a bespoke scoring system for the HBV ACLF patients, the HBV-ACLF score was developed. It is important to note that the performance of this etiology specific scoring system was significantly less accurate compared with the CLIF-C ACLF score confirming that this score is not be constrained by etiology. As the 1-year mortality rate was extremely low in the non-ACLF patients (49 patients; 8.3%), assessment of the CLIF-C AD score as a prognostic model for the non-ACLF patients^5^ is likely to be underpowered and therefore the data should be interpreted with caution. Nevertheless, the data show that the score performs significantly better than the MELD score at all the time points and does not significantly improve the performance of MELD-Na score. A bespoke HBV scoring system for the non-ACLF HBV cohort was developed from the data. Its performance was shown to be similar to that of CLIF-C AD score confirming its validity in the HBV non-ACLF population.

There are two further points that should be emphasized. Firstly, NUCs were not covered by nation wide medical insurance in China before 2009, the persistence of antiviral therapy for HBV was paid by the patient’s themselves. This can explain the reason why only a low percentage of enrolled patients (23.5%) were treated by NUCs before enrollment (patients’ data collected between 2005 and 2010). Secondly, a recent study^24^ from another HBV high endemic region in China has reported the rate of both flare-up and exacerbation of HBV were 35.8% in ACLF patients, while our HBV reactivation rate was 9.0%. Different definition about HBV reactivation and flare-up/exacerbation caused the significant gap in these two studies. HBV reactivation was defined as both ALT > 3NL and HBV-DNA > 100 U/L due to NUC resistance and cessation of the antiviral treatment in current study. However, In the study by Shi Y *et al*.’s study HBV ‘flare-up’ was defined as more than twice of the baseline value with HBV DNA positive within one month before admission and ‘exacerbation’ defined as ALT > 5NL with HBV DNA positive^24^.

There are still lack of clarity amongst Asian hepatologists in understanding whether acute decompensation (AD) of cirrhotic patients is related to precipitating factors or disease complications. In current study, 52% of HBV ACLF patients had new occurrence of decompensation (Table 4) which reflects that a precipitating event is the cause of AD in more than half the ACLF patients.

In current study multiple organ failure cirrhotic patients may include some chronic liver failure patients, However the liver specific multiple organ failure score (CLIF-OF) has clearly demonstrated that it can distinguish high mortality from low mortality patients not only in Eastern type (HBV) but also in Western type (Alcoholic) cirrhotic AD patients. This is the key point of this study to exhibit that a uniform diagnostic criterion could be applied for distinguishing ACLF from both HBV and alcoholic cirrhotic AD patients.

The study may be criticized because of its retrospective nature and the lack of a head to head comparative population with a non-HBV cohort. Nonetheless, because this study was based in a single hospital with access to all the patient records and biochemistry, there were very few missing data making the observations robust and increasing the validity of conclusions. Another potential criticism of this study is the management of the patients in a ward-based rather than ICU environment. It is possible that this may affect the results of the analysis but the similarities to the outcomes obtained and observations made in the CANONIC study, makes this is unlikely. Other groups have shown that the CLIF sequential organ failure assessment score (SOFA), which was first used in the CANONIC study to define ACLF, can be used to accurately classify patients further confirming the fidelity of the data and the validity of the observations from the present study^25^,^26^,^27^,^28^,^29^,^30^,^31^.

In summary, although ACLF in HBV patients (present study) and in patients with mainly alcohol related or hepatitis C virus (CANONIC study) exhibited differences in clinical features, the data in this study demonstrates that ACLF is a clinically and pathophysiologically distinct entity independent of the underlying etiology. First, age, previous decompensations and the presence or type of precipitating events were similar in the disease course and prognosis in both populations. Second, a systemic inflammatory (white cell count) response independently correlated with the presence and grade of ACLF and was an independent predictor of mortality. Third, the liver specific multiple organ failure score (CLIF-C OFs) had the equal diagnostic ability to differentiate ACLF from AD patients and the number of OFs determined short term mortality of the patients. Finally, the prognostic score designed in a non-HBV cohort was equally accurate for patients with HBV and could not be improved significantly increased by a bespoke disease specific score.

In conclusion, ACLF is by far the most frequent cause of death in patients with acute decompensation due to HBV infection, significantly more frequent than in patients with alcoholic and/or HCV infection. The data presented here confirm the validity of the CLIF criteria in the diagnosis and prognosis of patients with ACLF. The study also identified key clinical differences that should be the subject of future studies.

## Methods

### Study and patients characteristics

The study consisted in a retrospective analysis of 890 patients admitted to Ren Ji Hospital, Shanghai Jiao Tong University, from January 2005–December 2010 from a total of 3004 patients admitted to the hospital (Supplementary table S1). Patients with chronic hepatitis B virus infection and cirrhosis (hepatitis B surface antigen (HBsAg) +ve and antibody to hepatitis B core antigen (anti-HBc) +ve in the absence of other potential cause of cirrhosis; chronicity was defined by the presence of HBsAg +ve for > 6 months) who had either ascites, hepatic encephalopathy, variceal hemorrhage and/or bacterial infections, or other clinical, ultrasonography and/or laboratory data compatible with the diagnosis of cirrhosis were recruited in the analysis. The criterion of bacterial infection in current study was the same as CANONIC Study^4^ and included spontaneous bacterial peritonitis, spontaneous bacteremia (positive blood culture), urinary tract infection, pneumonia and cellulitis. Two hundred and sixty-four patients received an orthotopic (87%) or living donor (13%) liver transplant (LT) within 90-days following admission and in 95% of these patients the presence of cirrhosis was confirmed by liver histology. Thirteen patients had advanced fibrosis histologically. Informed consent had been obtained from all patients and/or their relatives about usage of their clinical and pathological data. The ethics committee of the Ren Ji Hospital approved the study and methods (Ethics Number EC (2014) 148K). Methods were carried out in accordance with the approved guidelines.

### Data collection

Data collection was performed in December 2013. Demographic data, prior history of a decompensation, main cause of admission, precipitating events (PE) associated with the acute decompensation or ACLF, other clinical and exploratory data, laboratory tests, mean arterial pressure, pulse oximetry, markers of HBV infection, and antiviral treatment given within 6-months prior to and during hospitalization were obtained from patient medical records or the hospital database. Clinical and laboratory data given in the article are those obtained at the time of admission into the hospital or, when indicated, at the time of diagnosis of ACLF. Survival rates at 28-days, 90-days, 6-months and 1-year following enrollment were obtained through patient medical records or by direct contact with the patients or their families.

### Diagnostic criteria for ‘reactivation’ of HBV infection

Reactivation of hepatitis B^32^,^33^,^34^ was diagnosed if patients presented with at least one of the following criteria:

1. Absolute increase in both HBV-DNA (>1000 copies/ml; [>200 IU/ml]) and ALT > 3 times normal in a patient on continuous treatment with nucleotide analogs (NUCs) due to the development of NUC resistance OR
2. An acute increase in HBV-DNA and ALT in a patient on continuous treatment with NUCs following cessation of the antiviral treatment OR
3. Acute increase in ALT associated with HBsAg reappearance in 2 consecutive tests 5 days apart and increase in HBV-DNA (>10^5^ copies/ml; [>2 × 10^4^ IU/ml]) in a patient with prior negative HBsAg and positive anti-HBc antibody.

### Diagnostic criteria of ACLF and prognostic scores

Definitions, diagnostic criteria and methodology used to analyze the study data were those proposed by the investigators of the CLIF Consortium in the CANONIC Study^3^ (Supplementary Figure S2). The term ACLF defines a syndrome characterized by AD of cirrhosis associated to organ failure (OF) and poor short-term probability of survival^3^,^5^. CLIF-C OFs was used for the diagnosis of organ failures (liver failure: serum bilirubin ≥12 mg/dl; renal failure: serum creatinine ≥2 mg/dl or renal support therapy; cerebral failure: grade III-IV hepatic encephalopathy; coagulation failure: INR ≥2.5; respiratory failure: PaO_2_/FiO_2_ ≤ 200 or SpO_2_/FiO_2_ ≤214; circulatory failure: vasoconstrictor requirements to maintain arterial pressure). In addition to organ failure, renal dysfunction (as defined by a serum creatinine of 1.5–1.9 mg/dl) and/or cerebral dysfunction (grade 1–2 hepatic encephalopathy) were also used for the diagnosis of ACLF in patients with single non-renal organ failure. (Supplementary Figure S2).

Based on the presence of organ failure and renal and/or cerebral dysfunction the following groups of patients were either excluded or included from the diagnosis of ACLF: 1. Excluded: (a) No organ failure; (b) Single non-renal organ failure without renal and/or cerebral dysfunction. Included: (A) Single renal failure (ACLF grade I, ACLF-1); (B) Single non-renal organ failure plus renal dysfunction and/or cerebral dysfunction (ACLF-1); (C) 2 organ failures (ACLF-2); (D) 3–6 OFs (ACLF-3)^4^.

CLIF-C ACLF score (based on CLIF-C OF score plus age and white blood cell count, WCC), CLIF-C AD score (based on serum creatinine, INR, serum sodium, age and WCC) were used to assess risk of mortality in patients with ACLF and AD^3^,^5^, respectively and compared to MELD^23^, and MELD-Sodium (MELD-Na) scores^21^.

An online application to compute CLIF Consortium scores and estimate the correspondingpredicteddeath rate at time ‘t’ is available at the CLIF-Consortium website: http://www.clifconsortium.com/.

### Statistics

All the data was analyzed at the Data Management Centre of the CLIF Consortium, Barcelona in line with the analysis of the previously published CANONIC study. All the variables used in statistical analysis were obtained at the time of hospital admission or, when indicated, at the time of diagnosis of ACLF. Data are presented as mean ± standard deviation for continuous parameters or frequencies and percentages for categorical variables. Mortality rates were estimated as transplant-free (patients who received a liver transplant were considered as lost to follow-up). Univariate analysis using Chi-square, Student’s t test or one way analysis of variance were performed to assess differences between groups or the association between all potential factors and outcomes. Two specific scores for HBV patients with and without ACLF (HBV-ACLF and HBV-AD scores) were estimated by fitting two Cox proportional-hazards models for 28-day and 1-year mortality, respectively. The initial model included all the risk factors found to be significantly associated with mortality in univariate analyses. Each score coefficients were based on the best independent sub-sets of predictors selected by means of the stepwise-forward method based on changes in model Likelihood Ratio (p-in = p-out = 0.05).

Harrell’s concordance index (C-index) was used to assess the discrimination ability of each severity score^35^.

Statistical comparison of C-index estimates was carried out for the main study time-points using the standardized normal approximation. A confirmatory analysis was carried out to assess the discrimination ability of the prognostic scores by estimating and comparing the corresponding Areas under the ROC curves (AUROCs). A univariate analysis was also carried out to identify the main baseline factors associated to the development of ACLF during the hospitalization in patients without ACLF at admission. A Logistic Regression model was fitted including all the significant potential predictors from this analysis. The best independent predictors of ACLF development in those without ACLF at presentation was selected by means of the stepwise-forward method based on changes in the model Likelihood Ratio (p-in = p-out = 0.05). In all statistical tests, the significance level was set at p < 0.05.

## Additional Information

**How to cite this article**: Li, H. *et al*. Characteristics, Diagnosis and Prognosis of Acute-on-Chronic Liver Failure in Cirrhosis Associated to Hepatitis B. *Sci. Rep*. **6**, 25487; doi: 10.1038/srep25487 (2016).

## Supplementary Material

Supplementary Information

## Figures and Tables

**Table 1:** Characteristics and mortality of patients with and without ACLF during the study period (admission plus hospitalization).

Characteristics	No ACLF (N = 590)	ACLF (N = 300)	*p*-value	ACLF grade I (N = 55)	ACLF grade II (N = 147)	ACLF grade III (N = 98)	*p*-value
**Age (y)**	51.8 ± 10.7	46.5 ± 11.3	<0.001	49.6 ± 11.3	44.8 ± 11.2	47.1 ± 11.0	<0.001
**Male sex**	450(76.3)	233(77.7)	0.641	41(74.6)	119(81.0)	73(74.5)	0.581
**Ascites**	482(81.7)	229(76.6)	0.072	42(76.4)	115(78.8)	72(73.5)	0.235
**Mean arterial pressure (mm Hg)**	87 ± 11	84 ± 18	0.011	87 ± 18	87 ± 15	78 ± 21	<0.001
**Cause of cirrhosis**			0.002				<0.001
HBV alone	544(92.3)	263(87.7)		47(85.6)	130(88.5)	86(87.7)	
HBV + Alcohol	36(6.1)	19(6.3)		4(7.2)	7(4.7)	8(8.2)	
HBV + other hepatitis virus	5(0.8)	12(4.0)		2(3.6)	9(6.1)	1(1.0)	
HBV + schistosomiasis	5(0.8)	6(2.0)		2(3.6)	1(0.7)	3(3.1)	
**HBV-DNA level (IU/ml):**							
≤100	97 (16.4)	60 (20.0)	0.069	7(12.7)	25(17.0)	28(28.6)	0.058
>100–2 × 10^4^	278 (47.1)	157 (52.3)		30(54.5)	75(51.0)	52(53.1)	
>2 × 10^4^–2 × 10^6^	176 (29.8)	68 (22.7)		13(23.6)	39(26.5)	16(16.3)	
>2 × 10^6^	39 (6.6)	15 (5.0)		5(9.1)	8(5.4)	2(2.0)	
**HbeAg positve**#	194(33.8)	99(35.2)	0.678	14(28.6)	53(36.3%	32(73.2%)	0.556
Treatment with NUCs*	114 (19.3)	95 (31.7)	<0.0001	10(20)	57(37.0)	28(29.2)	0.065
**Types of NUCs treatment before enrollment:**							
Lamivudinealone	67/114 (58.8)	48/95 (50.5)	0.233	4/10(40.0)	30/57(52.6)	14/28(50.0)	0.760
Entecaviralone	16/114 (14.0)	23/95 (24.2)	0.06	4/10 (40.0)	14/57 (24.6)	5/28 (17.9)	0.391
Adefoviralone	15/114 (13.2)	7/95 (7.4)	0.174	2/10 (20.0)	2/57 (3.5)	3/28 (10.7)	0.167
Telbivudinealone	3 /114(2.6)	2 /95(2.1)	0.803	0/10 (0.0)	1/57 (1.8)	1/28 (3.6)	0.703
Tenofoviralone	0 /114(0.0)	0/95 (0.0)	1.000	0/10 (0.0)	0/57 (0.0)	0/28 (0.0)	1.000
≥2 NUCs	13/114 (11.4)	15/95 (15.8)	0.354	0/10 (0.0)	10/57 (17.5)	5/28 (17.9)	0.161
**Potential precipitating events of ACLF**							
Bacterial infection***	35(5.9)	59(19.7)	<0.001	13(23.6)	22(15.1)	24(24.5)	<0.001
Gastrointestinal haemorrhage***	53(9.0)	23(7.7)	0.507	5(9.1)	6(4.1)	12(12.2)	0.131
Active alcoholism**	36(6.1)	30(10.0)	0.036	4(7.3)	18(12.2)	8(8.2)	0.087
HBV reactivation***	14(2.4)	27(9.0)	<0.001	5(9.1)	16(10.9)	6(6.1)	<0.001
Superimposed by hepatitis viruses	5(0.8)	9(3.0)	0.031	1(1.8)	7(4.8)	1(1.0)	0.182
Surgery**	7(1.2)	2(0.7)	0.705	0(0.0)	0(0.0)	2(2.0)	0.105
Hepatotoxic drugs or herbs**	8(1.4)	7(2.3)	0.284	1(1.8)	1(0.7)	5(5.1)	0.083
Portal vein thrombosis by CT/MRI***	50(8.5)	9(3.0)	0.002	3(5.5)	3(2.0)	3(3.1)	0.486
**Precipitating Events(PEs)**							
No PE	267(45.3)	135(45.0)	0.991	23(41.8)	75(51.0)	37(37.8)	0.253
1 PE	257(43.6)	132(44.0)		25(45.5)	60(40.8)	47(48.0)	
>1 PE	66(11.2)	33(11.0)		7(12.7)	12(8.2)	14(14.3)	
**Organ failure**							
Liver failure	46(7.8)	233(77.7)	<0.001	26(47.3)	129(87.8)	78(79.6)	<0.001
Kidney failure	0(0.0)	85(28.3)	<0.001	23(41.8)	16(10.9)	46(47.0)	<0.001
Cerebral failure	3(0.5)	71(23.7)	<0.001	1(1.8)	12(8.2)	58(59.2)	<0.001
Coagulation failure	58(9.8)	203(67.7)	<0.001	4(7.3)	120(81.6)	79(80.6)	<0.001
Circulation failure	2(0.3)	57(19.0)	<0.001	1(1.8)	11(7.5)	45(45.9)	<0.001
Lungs failure	0(0.0)	43(14.3)	<0.001	0(0.0)	6(4.1)	37(37.8)	<0.001
Renal dysfunction	2(0.4)	44(14.6)	<0.001	22(40.7)	14(9.9)	8(8.3)	<0.001
Cerebral dysfunction	17(2.9)	54(18.0)	<0.001	19(34.6)	28(19.1)	10(10.2)	<0.001
**Laboratory data**							
Hematocrit (%)	30 ± 7	30 ± 8	0.876	29 ± 8	31 ± 8	29 ± 8	0.218
Platelet count (×10^9^/L)	73 ± 56	85 ± 63	0.007	92 ± 79	80 ± 56	88 ± 64	0.020
Serum bilirubin (mg/dL)	5.1 ± 8.5	26.5 ± 16.3	<0.001	18.2 ± 17.8	29.4 ± 14.6	26.9 ± 16.2	<0.001
International normalized ratio	1.6 ± 0.6	3.2 ± 2.1	<0.001	1.8 ± 0.4	3.5 ± 2.6	3.5 ± 1.4	<0.001
Alanine aminotransferase (U/L)	75 ± 203	315 ± 589	<0.001	162 ± 281	327 ± 563	385 ± 730	<0.001
Aspartate aminotransferase (U/L)	88 ± 174	254 ± 451	<0.001	117 ± 103	292 ± 530	274 ± 430	<0.001
γ-Glutamyltransferase (U/L)	61 ± 71	70 ± 98	0.159	70 ± 99	70 ± 103	69 ± 89	0.491
Serum creatinine (mg/dL)	0.8 ± 0.2	1.5 ± 1.5	<0.001	1.9 ± 2.2	1.2 ± 1.1	1.9 ± 1.4	<0.001
Serum sodium (mmol/L)	136 ± 9	130 ± 7.5	<0.001	132 ± 6.2	130 ± 7	129 ± 9	<0.001
Leukocytecount (× 10^9^/L)	4.7 ± 3.3	10.3 ± 6.8	<0.001	8.1 ± 5.2	9.5 ± 5.3	12.8 ± 8.7	<0.001
**Previous decompensation**			0.002				0.015
No	243(41.2)	157(52.3)		28(50.9)	80(54.4)	49(50.0)	
Yes	347(58.8)	143(47.7)		27(49.1)	67(45.6)	49(50.0)	
**Mortality(LT-free)**							
28 days	15(2.6)	132(44.0)	<0.001	13(23.6)	60(40.8)	59(60.2)	<0.001
90 days	27(4.6)	150(50.0)	<0.001	19(34.6)	69(46.9)	62(63.3)	<0.001
180 days	34(5.8)	153(51.0)	<0.001	19(34.6)	70(47.6)	64(63.3)	<0.001
365 days	49(8.3)	155(51.7)	<0.001	21(38.2)	70(47.6)	64(63.3)	<0.001

*within 6 months prior admission; **within 3months prior admission; ***at admission; ^#^35 patients (19 patients had ACLF) didn’t have HbeAg test.

**Table 2:** Univariate analysis of ACLF development during hospitalization in patients without ACLF at admission.

Characteristics	Patients not developing ACLF N = 590	Patients developing ACLF N = 57	*p*-value
**Age (y)**	51.8 ± 10.7	51.8 ± 8.8	0.983
**Male sex**	450(76.3)	43(75.4)	0.888
Ascites*	482(81.7)	46(82.1)	0.994
**Mean arterial pressure (mm Hg)**	87 ± 11	85 ± 11	0.245
**Cause of cirrhosis**			0.025
HBV alone	544(92.8)	48(85.7)	
HBV + Alcohol	36(6.1)	5(8.9)	
HBV + other hepatitis virus	1(0.2)	0(0.0)	
HBV + schistosomiasis	5(0.9)	3(5.4)	
**HBV-DNA level (IU/ml):**			0.326
≤100	97(16.4)	12(21.1)	
>100–2 × 10^4^	278(47.1)	30(52.6)	
>2 × 10^4^–2 × 10^6^	176(29.8)	14(24.6)	
>2 × 10^6^	39(6.6)	1(1.8)	
**Potential precipitating events of ACLF**			
Bacterial infection	35(5.9)	11(19.6)	<0.001
Gastrointestinal haemorrhage	53(9.0)	7(12.3)	0.412
Active alcoholism**	36(6.1)	7(12.3)	0.074
HBV reactivation	14(2.4)	1(1.8)	0.767
Superimposed by hepatitis viruses	5(0.8)	0(0.0)	<0.001
Portal vein thrombosis by CT/MRI on admission*	50(8.5)	3(5.3)	0.554
Surgery**	7(1.2)	0(0.0)	<0.001
Hepatotoxic drugs or herbs**	8(1.4)	0(0.0)	<0.001
Physiological exhaustion**	6(1.0)	3(5.3)	0.043
**Precipitating Events(PEs)**			
No PE	267(45.3)	16(28.1)	0.043
1 PE	257(43.6)	32(56.1)	
>1 PE	66(11.2)	9(15.8)	
**Organ failures**			
Liver	46(7.8)	23(40.4)	<0.001
Kidney	0(0.0)	0(0.0)	–
Cerebral	3(0.5)	0(0.0)	0.589
Coagulation	58(9.8)	6(10.5)	0.867
Circulation	2(0.3)	1(1.8)	0.133
Lungs	0(0.0)	0(0.0)	–
Renal dysfunction	2(0.4)	1(1.9)	0.605
Cerebral dysfunction	17(2.9)	4(7.0)	0.092
**Laboratory data**			
Hematocrit (%)	30 ± 7	27 ± 6	0.001
Platelet count (×10^9^/L)	73 ± 56	87 ± 59	0.069
Serum bilirubin (mg/dL)	5.1 ± 8.5	14.5 ± ± 14.2	<0.001
International normalized ratio	1.6 ± 0.6	1.9 ± 0.6	<0.001
Alanine aminotransferase (U/L)	75 ± 203	134 ± 211	0.043
Aspartate aminotransferase (U/L)	88 ± 174	153 ± 195	0.011
γ-Glutamyltransferase (U/L)	61 ± 71	57 ± 77	0.751
Serum creatinine (mg/dL)	0.8 ± 0.2	0.8 ± 0.3	0.054
Serum sodium (mmol/L)	136 ± 9	129 ± 8	<0.001
Leukocyte count (×10^9^/L)	4.7 ± 3.3	8.6 ± 4.7	<0.001
**Previous decompensation**			0.696
No	243(41.2)	25(43.9)	
Yes	347(58.8)	32(56.1)	

*at admission; **within 3 months prior hospitalization.

**Table 3:** HBV-ACLFs and HBV-ADs comparing with c-index of CLIF-C ACLFs and CLIF-C ADs and with MELDs andMELD-Nas.

	C-index							
	28 days	P	90 days	P	180 days	P	360 days	p
**Patients with ACLF in the whole study**								
HBV-ACLFs	0.654 (0.604–0.705)		0.645 (0.596–0.694)		0.644 (0.595–0.693)		0.640 (0.591–0.688)	
CLIF-C ACLFs	0.704 (0.661–0.748)	0.023	0.685 (0.643–0.727)	0.057	0.687 (0.645–0.728)	0.041	0.682 (0.640–0.723)	0.046
MELDs	0.554 (0.497–0.610)	<0.001	0.543 (0.490–0.596)	<0.001	0.543 (0.491–0.595)	<0.001	0.540 (0.488–0.591)	<0.001
MELD-Nas	0.549 (0.493–0.605)	<0.001	0.541 (0.488–0.594)	<0.001	0.541 (0.488–0.594)	<0.001	0.537 (0.486–0.589)	<0.001
**Patients without ACLF in the whole study**								
HBV-ADs	0.737 (0.659–0.814)		0.716 (0.650–0.781)		0.720 (0.659–0.782)		0.721 (0.666–0.775)	
CLIF-C ADs	0.733 (0.662–0.803)	0.92	0.724 (0.663–0.784)	0.796	0.728 (0.671–0.784)	0.783	0.728 (0.677–0.779)	0.788
MELDs	0.667 (0.575–0.759)	0.08	0.653 (0.580–0.725)	0.056	0.657 (0.589–0.724)	0.042	0.639 (0.577–0.700)	0.003
MELD-Nas	0.719 (0.643–0.796)	0.653	0.710 (0.646–0.773)	0.886	0.701 (0.640–0.762)	0.54	0.682 (0.626–0.738)	0.164

**Table 4:** Differences of clinical characteristics between patients with ACLF included in the current study and patients from the CANONIC study.

Characteristics	Chinese Patients N = 300	CANONIC patients N = 417	*p*-value
Age (y)	46.5 ± 11.3	55.8 ± 11.7	<0.001
Male sex	233(77.7)	267(64.0)	<0.001
**Previous decompensation**			<0.001
No	157(52.3)	98(24.9)	
Yes	143(47.7)	295(75.1)	
**Ascites**	229(76.6)	324(78.5)	0.7346
**Mean arterial pressure(mmHg)**	84 ± 18	81 ± 13	0.0142
**Cause of cirrhosis**			
Alcohol,noHCV	–	233(58.4)	–
No Alcohol,HCV	–	59(14.8)	–
Alcohol+HCV	–	37(9.3)	–
Other Etiologies*	–	70(17.5)	–
HBV alone	263(87.7)	–	–
HBV+Alcohol	19(6.3)	–	–
HBV+other hepatitis virus	12(4.0)	–	–
HBV+schistosomiasis	6(2.0)	–	–
**Organ failures**			
Liver	233(77.7)	157(37.7)	<0.001
Kidney	85(28.3)	217(52.0)	<0.001
Cerebral	71(23.7)	94(22.5)	0.7925
Coagulation	203(67.7)	118(28.3)	<0.001
Circulation	57(19.0)	78(18.7)	0.9977
Lungs	43(14.3)	47(11.3)	0.2684
Renal dysfunction	44(14.7)	64(15.4)	0.801
Cerebral dysfunction	54(18.0)	130(31.3)	<0.001
**Laboratory data**			
Hematocrit(%)	30 ± 8	28 ± 9	0.0018
Leucocytes(×10^9^/L)	10.3 ± 6.8	9.5 ± 6.0	0.1033
Platelet count(×10^9^/L)	85 ± 63	103 ± 70	<0.001
Serum bilirubin(mg/dL)	27 ± 16	11 ± 11	<0.001
International normalized ratio	3.2 ± 2.1	2.0 ± 0.8	<0.001
Alanine aminotransferase(U/L)	315 ± 589	69 ± 122	<0.001
Aspartate aminotransferase(U/L)	254 ± 451	202 ± 991	0.3454
γ-Glutamyltransferase(U/L)	70 ± 98	154 ± 207	<0.001
Serum creatinine(mg/dL)	1.5 ± 1.5	2.0 ± 1.5	<0.001
Serum sodium(mmol/L)	130 ± 7	133.5 ± 7	<0.001
**Site of hospitalization**			<0.001
Intensive care unit	0(0.0)	98(23.6)	
Ward	300(100.0)	318(76.4)	
**ACLF grades:**			<0.001
ACLF-I	55(18.3)	214(51.3)	
ACLF-II	147(49.0)	147(35.3)	
ACLF-III	98(32.7)	56(13.4)	
**Mortality**			
28 days	129(43.0)	125(30.0)	<0.001
90 days	151(50.3)	178(42.7)	0.0624
180 days	154(51.3)	204(48.9)	0.6358
365 days	156(52.0)	228(54.7)	0.4709

^*^Hepatitis B-associated cirrhosis in 21 patients (5.2%).

The CANONIC data is reproduced with permission from Moreau *et al*. Gastro2013.
