# Supplementary material for: Characteristics, Diagnosis and Prognosis of Acute-on-Chronic Liver Failure in Cirrhosis Associated to Hepatitis B
Source: Sci Rep. 2016 May 5;6:25487. doi: 10.1038/srep25487 (PMC4857102; doi:10.1038/srep25487)
Supplement: Supplementary Information [file srep25487-s1.pdf]

Supplementary Appendix

Characteristics, Diagnosis and Prognosis of Acute-on-Chronic Liver Failure in Cirrhosis Associated to Hepatitis B.

Hai Li, Liu-Ying Chen, Nan-nan Zhang, Shu-Ting Li, Bo Zeng, Marco Pavesi, Àlex Navarro, Rajeshwar P Mookerjee, Qian Xia, Feng Xue, Xiong Ma, Jing Hua, Li Sheng, De-kai Qiu, Qing Xie, Graham R Foster, Geoffrey Dusheiko, Richard Moreau, Pere Gines, Vicente Arroyo, Rajiv Jalan.

Table of Contents

**Table/Figure**	**Title**	**Page**
Supplementary Table S1	Characteristics of Patients at hospital admission.	3-5
Supplementary Table S2	Characteristics and prognosis of patients with ACLF with and without prior history of AD.	6-8
Supplementary Table S3	Clinical characteristics and mortality according to the presence/absence or type of precipitating events in patients with ACLF	9-10
Supplementary Figure S1	The CLIF-Organ Failure score system.	11

Supplementary Table S1. Characteristics of Patients at hospital admission.

Age(y)	50.0±11.2
Male sex	683(76.7)
**Cause of cirrhosis**	
HBValone	807(90.7)
HBV+Alcohol	55(6.2)
HBV+other hepatitis virus	17(1.9)
HBV+schistosomiasis	11(1.2)
**HBV-DNA level(IU/ml)**	
≤100	157(17.6)
>100-2x10^4	435(48.9)
>2x10^4-2x10^6	244(27.4)
>2x10^6	54(6.1)
Treatment with NUCs*	209(23.5)
**Cause of hospitalization at enrolment**	
Ascites	506(56.9)
Hepatic Encephalopathy	103(11.8)
Bacterial infections	40(4.4)
GI-bleeding	237(26.5)
Other cause	4(0.4)
**Potential precipitating events(PE) of ACLF**	
No PE’s	402(45.2)
At least one PE	389(43.7)
>1PE	99(11.1)
Bacterial infection	94(10.6)

Gastrointestinal hemorrhage	67(7.5)
Active alcoholism**	64(7.2)
HBV reactivation	41(4.6)
others	151(16.9)
**Previous decompensation**	
No	400(44.9)
Yes	490(55.1)
**Site of hospitalization at enrolment**	
Intensive care unit	0(0.0)
Ward	890(100.0)
**Laboratory data**	
Hematocrit(%)	30±7
Platelet count(x 10^9/L)	78±59
Serum bilirubin(mg/dL)	11.8±14.9
International normalized ratio	2.0±1.2
Alanine aminotransferase(U/L)	156±394
Aspartate aminotransferase(U/L)	139±293
γ-Glutamyltransferase(U/L)	64±79
Serum creatinine(mg/dL)	1.0±0.9
Serum sodium(mmol/L)	134±9
Leukocyte count(x10^9/L)	6.4±4.9
MELD score at enrolment	20.2±9.4
MELD na score at enrolment	22.6±9.3
Liver failure	268(30.1)
Kidney failure	89(10.0)
Cerebral failure	46(5.2)
Coagulation failure	232(26.1)

Respiration failure	11(1.2)
Circulation failure	21(2.4)
No organ ailure	510(57.3)
1 organ failure	180(20.2)
>1 organ failure	200(22.5)
Renal dysfunction	46(5.3)
Cerebral dysfunction	74(8.3)
**ACLF**	243(27.3)
GradeI	45(5.1)
GradeII	132(14.8)
GradeIII	66(7.4)

Data are mean±Standard Deviation or n(%)
*within 6months prior to enrolment
**within 3 months prior to hospitalization

Supplementary Table S2. Characteristics and prognosis of patients with ACLF with and without prior history of AD.

Characteristics	Any prior AD (n=143)	No prior AD (n=157)	*p*-value
Age(y)	46.9±10.4	46.1±12.0	0.518
Male sex	102(71.3)	131(83.4)	0.012
Ascites	110(76.9)	119(76.3)	0.896
**Cause of cirrhosis**			0.243
HBV alone	120(86.3)	143(91.1)	
HBV+Alcohol	13(9.4)	6(3.8)	
HBV+other hepatitis virus	4(2.9)	4(2.6)	
HBV+schistosomiasis	2(1.4)	4(2.6)	
**HBV-DNA level(IU/ml)**			
≤100	27(18.9)	33(21.0)	0.899
>100-$2x10^4$	74(51.7)	83(52.9)	
>$2x10^4$-$2x10^6$	35(24.5)	33(21.0)	
>$2x10^6$	7(4.9)	8(5.1)	
Treatment with NUCs*	46(32.2)	49(31.2)	0.859
**Potential precipitating events of ACLF**			
Bacterial infection	31(21.7)	28(18.0)	0.418
Gastrointestinal haemorrhage	19(13.3)	4(2.6)	<0.001
Active alcoholism**	14(9.8)	16(10.2)	0.908
HBV reactivation	19(13.3)	8(5.1)	0.013
others	20(14)	20(14)	1.000
No precipitating events	51(35.7)	84(53.5)	0.005
At least one precipitating event	71(49.7)	61(38.9)	

>1 precipitating events	21(14.7)	12(7.6)	
**Organ failures**			
Liver	102(72.3)	131(84.5)	0.011
Kidney	44(31.0)	41(26.5)	0.388
Cerebral	37(25.9)	34(21.7)	0.391
Coagulation	91(64.1)	112(72.3)	0.130
Circulation	35(24.5)	22(14.0)	0.021
Lungs	21(14.7)	22(11.4)	0.868
Renal dysfunction	21(15.1)	23(15.1)	0.996
Cerebral dysfunction	28(19.6)	29(18.5)	0.807
**ACLF**			0.197
GradeI	25(18.1)	20(13.4)	
GradeII	55(38.5	77(49.0)	
GradeIII	31(22.5)	35(22.3)	
**Laboratory data**			
Leukocyte count(x10^9/L)	10.4±7.7	10.3±6.0	0.879
Platelet count(x10^9/L)	81±68	88±58.8	0.335
Serum bilirubin(mg/dL)	23.6±15.9	29.2±16.3	0.003
International normalized ratio	3.1±1.9	3.3±2.2	0.322
Alanine aminotransferase(U/L)	241±501	382±653	0.036
Aspartate aminotransferase(U/L)	240±505	266±398	0.627
γ-Glutamyltransferase(U/L)	54±54	83±122	0.007
Serum creatinine(mg/dL)	1.6±1.7	1.5±1.3	0.007
**Mortality**			
28 days	66(46.2)	66(42.0)	0.473
90 days	74(51.8)	76(48.4)	0.563
180 days	77(53.9)	76(48.4)	0.347

365 days	78(54.6)	77(49.0)	0.341

Data are mean±Standard Deviation or n(%)
*within 6 months prior to enrolment
**within 3 months prior to hospitalization

Supplementary Table S3. Clinical characteristics and mortality according to the presence/absence or type of precipitating events in patients with ACLF

Characteristics	HBV reactivation<br>N=27	Other Precipitating event<br>N=81	No Precipitating event but HBV DNA>500<br>N=155	No Precipitating event and HBV DNA ≤500<br>N=37	*p*-value
Age(y)	41.5±8.6	48.3±10.7	45.7±11.5	49.1±12.3	0.018
Male sex	21(77.8)	65(80.3)	117(75.5)	30(81.1)	0.805
**Previous decompensation**					0.037
No	8(29.6)	39(48.2)	87(56.1)	23(62.2)	
Yes	19(70.4)	42(51.9)	68(43.9)	14(37.8)	
Ascites	22(81.5)	61(75.3)	136(87.7)	29(78.4)	0.096
Mean arterial pressure(mmHg)	95±15	85±15	85±13	88.7±14.9	0.002
**Organ failures**					
Liver	25(92.6)	51(63.0)	121(78.1)	25(67.6)	0.007
Kidney	6(22.2)	32(39.5)	41(26.5)	10(27.0)	0.147
Cerebral	4(14.8)	5(6.2)	22(14.2)	12(32.4)	0.003
Coagulation	19(70.4)	42(51.9)	90(58.1)	23(62.2)	0.360
Circulation	2(7.4)	9(11.1)	7(4.5)	1(2.7)	0.183
Lungs	1(3.7)	3(3.7)	4(2.6)	3(8.1)	0.460
Renal dysfunction	3(11.5)	13(16.9)	24(15.8)	4(11.1)	0.813
Cerebral dysfunction	9(33.3)	20(24.7)	24(15.5)	4(10.8)	0.043
**Laboratory data**					
Hematocrit(%)	36±7	29±7	29±8	31±9	0.001
Leucocytes	10.5±5.9	10.6±6.9	8.7±4.9	10.7±5.5	0.037
Platelet count($\times 10^9$/L)	86±42	91±74	86±60	86±60	0.937
Serum bilirubin(mg/dL)	28±10	23±17	27±17	20±13	0.040
International normalized ratio	3.3±1.3	2.6±1.1	2.9±1.4	3.7±3.1	0.008
Alanine aminotransferase(U/L)	508±412	223±497	336±669	272±423	0.149
Aspartate aminotransferase(U/L)	368±386	204±399	234±447	257±416	0.403
γ-Glutamyltransferase(U/L)	86±84	83±122	64±81	51±47	0.234
Serum creatinine(mg/dL)	1.6±2.8	1.5±1.2	1.3±1.3	1.6±1.4	0.561

Serum sodium(mmol/L)	130±7	131±8	129±7	131±7.7	0.105
**ACLF grades:**					0.047
ACLF-I	5(18.5)	17(21.0)	28(18.1)	5(13.5)	
ACLF-II	16(59.3)	31(38.3)	86(55.5)	14(37.8)	
ACLF-III	6(22.2)	33(40.7)	41(26.5)	18(48.7)	
**Mortality**					
28 days	12(44.4)	39(48.2)	56(36.1)	22(59.5)	0.047
90 days	13(48.2)	46(56.8)	69(44.5)	23(62.2)	0.134
180 days	13(48.2)	47(58.0)	71(45.8)	23(62.2)	0.158
365 days	13(48.2)	47(58.0)	73(47.1)	23(62.2)	0.225

Data are mean±Standard Deviation or n(%)

Supplementary Figure S1. The CLIF-organ failure score system

**Organ/system**	**Subscore = 1**	**Subscore = 2**	**Subscore = 3**
**Liver**	Bilirubin <6 mg/dl	Bilirubin ≥6 mg/dl and <12 mg/dl	Bilirubin ≥12 mg/dl
**Kidney**	Creatinine <2 mg/dl	Creatinine ≥2mg/dl and <3.5 mg/dl	Creatinine ≥3.5 mg/dl or renal replacement
**Brain (West-Haven grade for HE)**	Grade 0	Grade 1-2	Grade 3-4
**Coagulation**	INR <2.0	INR ≥2.0 and <2.5	INR ≥2.5
**Circulatory**	MAP ≥70 mmHg	MAP <70 mmHg	Use of vasopressors
**Respiratory PaO2/FiO2 or SpO2/FiO**	>300 or >357	≤300 and >200 or >214 and ≤357	≤200 or ≤214
